# The Impact of Yes-Associated Protein 1 (YAP1) Expression Patterns in Locally Advanced Breast Cancer: Associations with Pathological Response and Tumor Features

**DOI:** 10.3390/medicina61071297

**Published:** 2025-07-18

**Authors:** Osman Erinc, Sabin Goktas Aydin, Taskin Erkinuresin, Ozgur Yilmaz, Ahmet Aydin, Sevinc Dagistanli, Murat Akarsu

**Affiliations:** 1 Department of Internal Medicine, Kanuni Sultan Suleyman Training and Research Hospital, 34303 Istanbul, Turkey; dr_ozguryilmaz@hotmail.com (O.Y.); muratakarsu79@gmail.com (M.A.); 2 Department of Medical Oncology, Kanuni Sultan Suleyman Training and Research Hospital, 34303 Istanbul, Turkey; 3 Department of Pathology, Kanuni Sultan Suleyman Training and Research Hospital, 34303 Istanbul, Turkey; erkinuresin@hotmail.com; 4 Department of Internal Medicine, Medipol University Hospital, 34214 Istanbul, Turkey; 5 Department of General Surgery, Kanuni Sultan Suleyman Training and Research Hospital, 34303 Istanbul, Turkey

**Keywords:** YAP1, locally advanced breast cancer, neoadjuvant chemotherapy, axillary pCR, HER-2

## Abstract

*Background and Objectives*: The Hippo pathway, via Yes-associated protein 1 (YAP1), regulates cell proliferation, apoptosis, and tissue regeneration. Aberrant YAP1 activation is linked to tumor progression and immune evasion in various cancers, including breast carcinoma, despite conflicting evidence on its prognostic value. Preclinical studies have explored drugs targeting YAP1–TEAD interactions, but therapeutic application is limited. *Materials and Methods*: This study included 50 patients with locally advanced breast cancer, who were assessed by a multidisciplinary tumor board and underwent neoadjuvant treatment per tumor subtype and clinical guidelines. Eligibility required both pre-treatment core biopsy and post-treatment surgical resection samples. Due to the absence of residual tumor in some patients achieving complete pathological response, post-treatment tissue was available and analyzable in 30 patients. YAP1 expression was evaluated immunohistochemically for nuclear and cytoplasmic staining patterns. ROC analysis identified a cutoff for YAP1 expression, defining tumors with ≥70% nuclear and ≥80% cytoplasmic staining. *Results*: YAP1 expression had a significant relationship with tumor subtype (*p* = 0.001), being most frequent in HER-2-positive tumors (55.6%) and least frequent in luminal tumors (11.1%). YAP1 positivity significantly predicted axillary pathological complete response (pCR) (*p* = 0.01). In YAP1-positive patients, 77.8% achieved axillary pCR compared to 31.7% in YAP1-negative patients, though the YAP1 status and breast pCR association were insignificant (*p* = 0.07). The Mann–Whitney U test indicated that higher Ki-67 values were significantly associated with positive YAP1 expression (*p* = 0.028). In contrast, there was no association between ER, PR status, age, and tumor size. Following treatment, there was a statistically significant change in YAP1 expression, with nuclear staining decreasing (*p* = 0.004) while cytoplasmic staining increased (*p* = 0.002). YAP1 was significantly linked to axillary pCR, HER-2 status, and Ki-67. *Conclusions*: Post treatment, nuclear YAP1 decreased, whereas cytoplasmic expression increased, showing a localization shift. These results suggest that YAP1 may predict treatment response and become a future therapeutic target.

## 1. Introduction

Breast cancer is the most frequently diagnosed cancer and a leading cause of cancer-related death in women, accounting for one in four cancer cases and one in six cancer deaths [1]. Locally advanced breast cancer (LABC), a heterogeneous group defined by large tumors, regional lymph node involvement, or invasion of the chest wall or skin without distant metastasis, poses distinct clinical challenges [2]. Its standard management involves neoadjuvant chemotherapy (NAC), which aims to downstage the tumor, enable breast-conserving surgery, and assess early treatment response [3]. Achieving pathological complete response (pCR) after NAC correlates with better disease-free and overall survival, especially in triple-negative and HER-2 positive subtypes [4,5]; however, many patients respond poorly to NAC, resulting in worse outcomes [6]. Therefore, identifying reliable predictive and prognostic biomarkers remains essential to guide therapy and improve prognosis.

The Hippo signaling pathway, a key regulator of organ size and tissue homeostasis, has emerged as a critical player in cancer biology due to its involvement in proliferation, survival, and therapeutic resistance [7,8]. At the center of this pathway is Yes-associated protein-1 (YAP1), a transcriptional coactivator that promotes oncogenic transcriptional programs when translocated into the nucleus via interaction with TEAD transcription factors [7]. Aberrant activation of YAP1 has been implicated in the initiation and progression of various cancers, including lung, mesothelioma, gastric, and colorectal cancers, where it is associated with poor prognosis and resistance to targeted therapies [9,10,11,12,13].

Beyond its oncogenic role, YAP1 has shown predictive value in neoadjuvant settings; its overexpression has been linked to worse outcomes in esophageal and prostate cancers following preoperative treatment [14,15]. These findings position YAP1 as a pan-cancer marker of treatment resistance and progression.

In breast cancer, YAP1 expression is particularly enriched in high-grade, hormone receptor-negative tumors, including triple-negative breast cancer (TNBC) [16]. YAP1 contributes to chemoresistance by modulating anti-apoptotic pathways, drug efflux mechanisms, and cancer stem cell maintenance [16,17,18]. Inhibition of YAP1 reverses taxane resistance in TNBC models, underscoring its role in sustaining drug tolerance [17]. The upstream WISP1–YAP–TEAD4 axis has also been shown to drive chemoresistance, with its inhibition resulting in YAP inactivation and reduced tumor growth [19]. Additionally, YAP1-driven transcriptional responses may enable tumor cells to adapt to chemotherapy-induced stress, particularly in the neoadjuvant context [20].

However, conflicting findings exist regarding the role of YAP1 in breast cancer. Several studies have reported that nuclear YAP1 expression correlates with poor prognosis and chemoresistance. Cha et al. demonstrated that high nuclear YAP1 expression was independently associated with worse distant metastasis-free survival in patients with triple-negative breast cancer [21]. Similarly, analyses of genomic data from over 2000 breast cancer samples revealed that elevated YAP1 expression predicted significantly poorer 15-year survival, particularly in basal (ER-negative) subtypes [22]. In contrast, other studies have suggested that cytoplasmic YAP1 may exert tumor-suppressive effects, especially in luminal subtypes. Cao et al. reported that cytoplasmic—but not nuclear—YAP1 expression was associated with improved disease-free survival in luminal A breast cancer [23]. Additionally, a randomized controlled study indicated that low YAP1 mRNA expression is an independent prognostic factor for recurrence in the less aggressive luminal A breast cancer subgroup and may contribute to progression [24]. Despite these insights, limited data exist regarding the dynamic changes in YAP1 subcellular localization before and after NAC.

On the other hand, novel strategies aimed at modulating Hippo pathway activity or disrupting YAP–TEAD interactions are currently under investigation and may offer a path toward personalized treatment approaches in aggressive breast cancer subtypes [25,26].

Given these contexts, this study aimed to evaluate the dynamic changes in YAP1 expression patterns in patients with LABC who received NAC and to investigate its association with clinicopathologic features and pathological treatment response. Specifically, the study assessed both pre- and post-NAC YAP1 expression levels to explore the potential impact of chemotherapy on YAP1 dynamics.

## 2. Materials and Methods

### 2.1. Study Design and Population

Seventy-three patients with LABC who were evaluated by a multidisciplinary tumor board and scheduled to receive neoadjuvant treatment and underwent surgery between 1 January 2023 and 1 January 2025 were initially screened. Patients were eligible for inclusion if they had histologically confirmed LABC and had received NAC based on tumor subtype and current clinical guidelines. Additional inclusion criteria required the availability of both pre-treatment core needle biopsy and post-treatment surgical resection specimens, along with complete clinical, radiological, and pathological data, including follow-up information. Patients were excluded if they had evidence of distant metastasis (stage IV disease) at diagnosis, lacked either pre- or post-treatment tissue samples, had incomplete clinical or pathological records, received neoadjuvant endocrine therapy or radiotherapy alone without chemotherapy, were clinically node-negative at diagnosis, had any prior history of cancer, or were undergoing immunosuppressive treatment.

Data were gathered from patient charts, including their age, histopathological type and molecular subtype, tumor location, initial and postoperative Ki-67 index, and the NAC regimen. All patients underwent surgery and analysis of surgical specimens following NAC.

The radiological staging was evaluated by the American Joint Committee on Cancer/Union for International Cancer Control version 8 [2].

Pre-treatment core biopsy specimens were available and assessed for 50 patients who were enrolled in the study. Out of these 50 patients, post-NAC surgical specimens were accessible for only 30 patients due to pCR (*n* = 11) or near-complete response (*n* = 9).

### 2.2. Neoadjuvant Chemotherapy Protocol

The patients in the luminal group were treated with four cycles of adriamycin (60 mg/m^2^) combined with cyclophosphamide (600 mg/m^2^) (AC/EC) every two weeks, followed by 12 cycles of paclitaxel (80 mg/m^2^) administered weekly. Patients whose tumors were HER-2-positive received AC/EC followed by docetaxel with dual blockage (trastuzumab 8 mg/kg loading dose, followed by 6 mg/kg; pertuzumab 840 mg loading dose, followed by 420 mg; every three weeks). TNBC patients received four cycles of AC every two weeks, followed by 12 cycles of paclitaxel (80 mg/m^2^) with or without carboplatin 2 AUC on a weekly basis.

### 2.3. Pathological Evaluation

The histopathological classification was based on immunohistochemistry (IHC). Tumors were considered luminal if they were HER-2-negative and estrogen receptor (ER)-positive. Luminal A and B subtypes were distinguished by Ki-67 index, with luminal A defined as Ki-67 < 20% and luminal B as Ki-67 ≥ 20%. HER-2 positivity was assigned to cases with IHC 3+ or confirmed gene amplification by SISH. The Miller-Payne regression score assessed the pCR in histopathological specimens, where a pCR was characterized by the absence of residual invasive cancer in breast tissue, corresponding to the Miller-Payne score [27].

### 2.4. Immunohistochemical Evaluation of YAP1 Expression

Formalin-fixed, paraffin-embedded breast carcinoma tissue samples—including both tru-cut biopsies and post-neoadjuvant resection specimens—were sectioned at 3 μm thickness. After incubation at 60 °C for 1 h, slides were deparaffinized with xylene and rehydrated through graded ethanol series. Antigen retrieval was performed using a 1:10 diluted citrate buffer (pH 6.0) in a PT module. Endogenous peroxidase activity was blocked using 3% hydrogen peroxide for 10 min, followed by protein blocking. Sections were incubated with primary anti-YAP1 antibody (Bio SB, Inc., BSB-146, Santa Barbara, CA, USA) (1:50 dilution) for 2 h at room temperature. Detection was achieved using a polymer-based detection system with enhancer and HRP polymer steps, and visualization was performed using 3,3′-diaminobenzidine (DAB) chromogen (TA-125-HA, Thermo Scientific, Carlsbad, CA, USA). Slides were counterstained with hematoxylin (HHS32, Sigma, Roedermark, Germany), dehydrated, and mounted with Entellan. All immunohistochemical staining was carried out using the Sequenza automated IHC system (Sequenza Immunostaining Center Each 73300001, Shandon, China/Thermo Scientific, Carlsbad, CA, USA).

**Figure 1:**
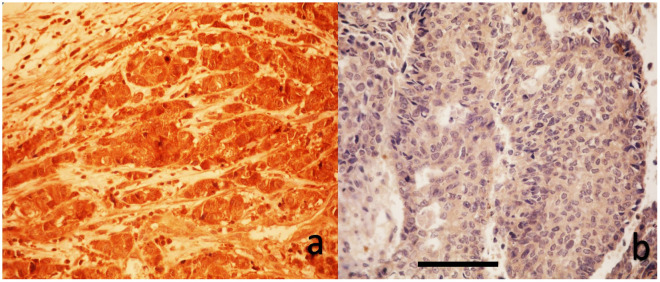
Immunohistochemical analysis of cytoplasmic YAP1 expression in breast carcinoma tissues. (**a**) High cytoplasmic YAP1 staining. (**b**) Weak cytoplasmic YAP1 staining (DAB with hematoxylin counterstain, original magnification 40×, scale bar represents 100 μm).

**Figure 2:**
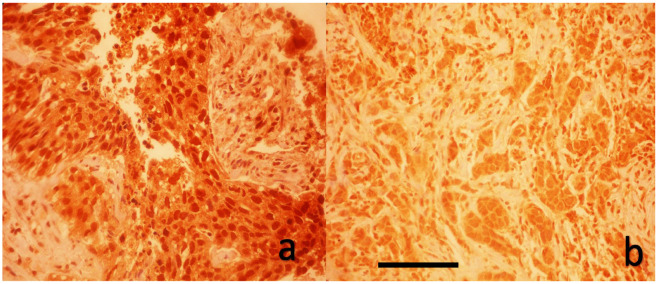
Immunohistochemical analysis of nuclear YAP1 expression in breast carcinoma tissues. (**a**) High nuclear YAP1 staining. (**b**) Weak nuclear YAP1 staining (DAB with hematoxylin counterstain, original magnification ×40, scale bar represents 100 μm).

All YAP1 immunohistochemical slides were evaluated by a single experienced breast pathologist who was blinded to clinical data. Due to resource constraints, inter-observer assessment was not performed. As shown in Figure 1 and Figure 2, a blinded pathologist evaluated YAP1 expression under 400× magnification. Both nuclear and cytoplasmic staining were assessed, with internal control based on the staining intensity of myoepithelial and luminal cells within terminal duct lobular units.

The AUC for nuclear expression was 0.569 (95% CI: 0.405–0.733; *p* = 0.411), and for cytoplasmic expression was 0.483 (95% CI: 0.318–0.649; *p* = 0.843). Because univariate ROC curves for baseline YAP1 percentages were not statistically significant (AUC < 0.60), we used the closest-to-(0,1) Youden method within a bivariate model (YAP1 + HER-2) to identify practical cutoffs that jointly maximized sensitivity for axillary pCR while limiting false positives. Consequently, tumors with ≥70% nuclear and ≥80% cytoplasmic staining were classified as YAP1-positive. Similar thresholds have been used in prior studies (28). Due to the limited availability of post-treatment tissue in cases of complete or near-complete pathological response, ROC-based cutoff determination was not feasible for post-treatment specimens. Therefore, post-treatment YAP1 expression data were analyzed as continuous variables without categorization.

### 2.5. Statistical Analysis

All statistical analyses were performed using IBM SPSS Statistics version 24 (SPSS Inc., Chicago, IL, USA). Descriptive statistics were used to summarize demographic and clinicopathological data. The distribution of continuous variables was assessed using the Shapiro–Wilk test. Since none of the continuous variables followed a normal distribution, non-parametric tests were applied for all relevant analyses. Associations between categorical variables were evaluated using Pearson’s chi-square test/Fisher’s exact test. The Mann–Whitney U test was used to compare continuous variables between independent groups. Analyses based on pre-treatment YAP1 expression and its association with clinical and pathological features and pCR were performed on the entire cohort (*n* = 50). Pre- and post-treatment changes in YAP1 expression were assessed using the Wilcoxon signed-rank test for paired samples. Paired comparisons of pre- versus post-NAC YAP1 expression used only those patients with both pre- and post-treatment specimens (N = 30). A receiver operating characteristic (ROC) curve analysis was conducted to define optimal cutoff values for YAP1 expression predicting the pCR status in breast and axillary tissue. A two-tailed *p*-value of <0.05 was considered statistically significant.

## 3. Results

A total of 50 patients with LABC were included, with a median age of 49.5 years (range, 24–72). Among them, 24 (48%) were premenopausal; 27 patients (54.0%) had hormone receptor (HR)-positive/HER-2-negative tumors (luminal type). Seven patients (14.0%) exhibited co-expression of hormone receptors and HER-2 (HR-positive/HER-2-positive), seven patients (14.0%) had HER-2-enriched tumors (HER-2-positive/HR-negative), while nine patients (18.0%) were classified as TNBC (Table 1).

In our cohort, YAP1 positivity was observed in 1 out of 27 patients (3.7%) with HR-positive/HER-2-negative tumors (luminal type). In contrast, YAP1 expression was detected in 5 out of 7 patients (71.4%) with HR-positive/HER-2-positive tumors and in 1 out of 7 patients (14.3%) with HER-2-enriched tumors. Among patients with triple-negative breast cancer, 2 out of 9 patients (22.2%) exhibited YAP1 positivity (*p* = 0.001). Pre-treatment YAP1 expression was present in 6 HER-2-positive and 3 HER-2-negative tumors, while none of the HER-2-low tumors showed YAP1 positivity (*p* = 0.01) (Table 2).

Following NAC, 20 patients (40%) achieved axillary pCR and 11 achieved breast pCR. The post-NAC final pathology report revealed that 25 (50.0%) had lymphatic invasion and 23 (46.0%) had vascular invasion.

YAP1 positivity significantly predicted axillary pCR (OR = 7.54, 95% CI: 1.37–41.41; *p* = 0.01). In YAP1-positive patients, 77.8% achieved axillary pCR compared to 31.7% in YAP1-negative patients, though the YAP1 status and breast pCR association were insignificant.

As shown in Table 2, among patients with YAP1-positive tumors, 4 out of 9 (44.4%) achieved breast pCR, compared to 7 out of 41 (17.1%) in the YAP1-negative group, indicating a higher but not statistically significant pCR rate in YAP1-positive tumors (OR = 3.89, 95% CI: 0.83–18.24; *p* = 0.07).

Breast pCR was achieved in 8 premenopausal and 3 postmenopausal patients, while 16 and 23 patients in each group, respectively, did not achieve pCR (*p* = 0.06) (Table 1).

Lymph node invasion was present in 23 of YAP1-negative patients (62.2%) and in 2 of YAP1-positive patients (22.2%) (*p* = 0.03). Vascular invasion was observed in 22 of YAP1-negative patients (59.5%) and in 1 of YAP1-positive patients (11.1%) (*p* = 0.009).

As expected, breast pCR rates significantly differed among breast cancer subtypes (*p* = 0.001). Among patients with a luminal tumor type, only 1 out of 27 (3.7%) achieved pCR, whereas higher pCR rates were observed in the luminal/HER-2 co-expressing (5/7; 71.4%), HER-2 only (3/7; 42.9%), and triple-negative (2/9; 22.2%) subtypes.

The Mann–Whitney U test indicated that higher Ki-67 values were significantly associated with positive pre-NAC YAP1 expression (*p* = 0.028). In contrast, there was no association between ER, progesterone receptor (PR) status, and tumor size (all *p* values > 0.05) (Table 2). Pre-NAC YAP 1 expression also was not associated with pre-T (*p* = 0.8) and N status (*p* = 0.6).

**Figure 3:**
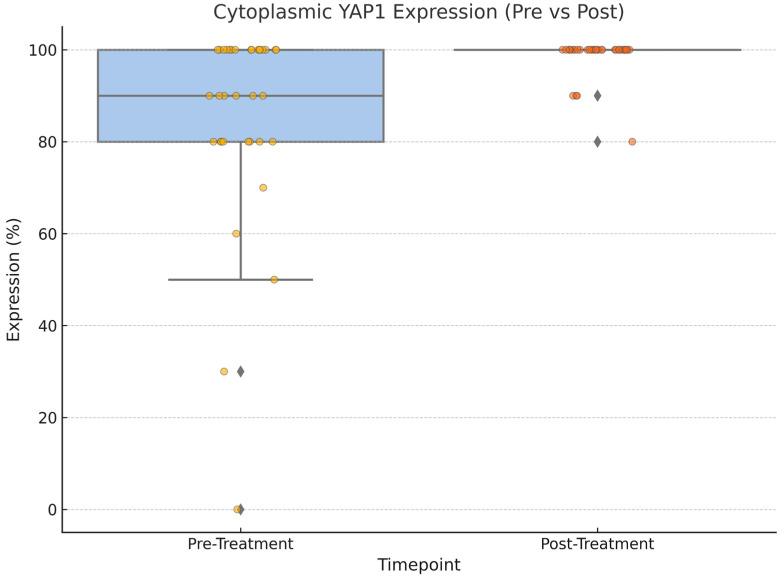
Cytoplasmic YAP1 expression before and after neoadjuvant chemotherapy. A significant increase was observed post treatment (Wilcoxon signed-rank test, *p* = 0.002).

**Figure 4:**
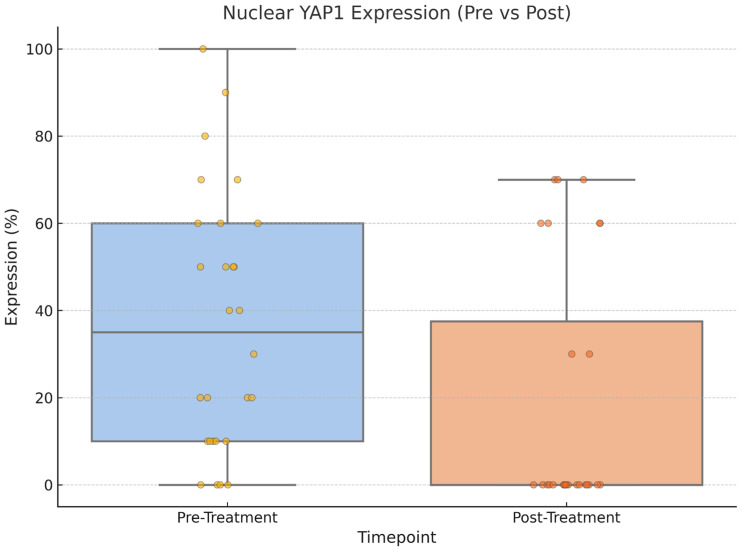
Nuclear YAP1 expression before and after neoadjuvant chemotherapy. A significant decrease was observed post treatment (Wilcoxon signed-rank test, *p* = 0.004).

**Figure 5:**
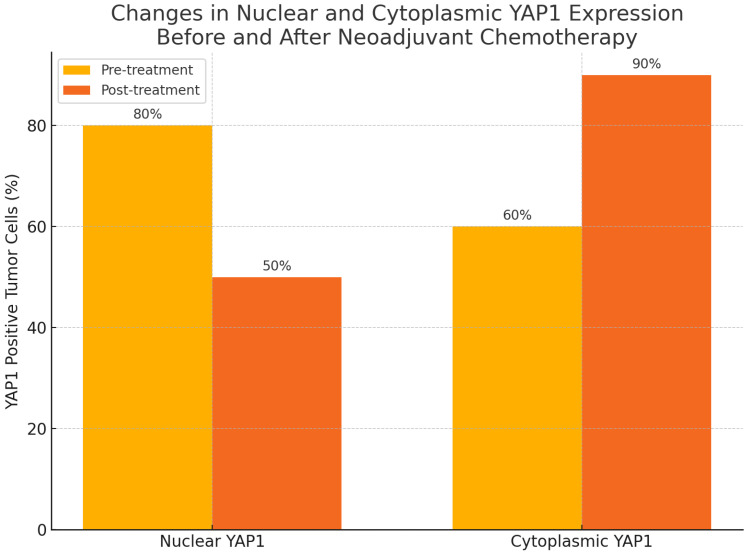
Changes in nuclear and cytoplasmic YAP1 expression before and after neoadjuvant chemotherapy.

Following treatment, there was a statistically significant change in YAP1 expression, with nuclear staining decreasing (*p* = 0.004) while cytoplasmic staining increased (*p* = 0.002), as shown in Figure 3, Figure 4 and Figure 5. Post-NAC YAP1 expression did not show significant correlation with ER, PR, or Ki-67 (all *p* values > 0.1).

## 4. Discussion

YAP1 is a key protein in the Hippo pathway that supports cancer growth and survival, particularly in aggressive breast cancers like triple-negative tumors [7,8,21]. Its increased expression is linked to poor response to chemotherapy, making it a potential marker for treatment resistance and a target for new therapies [16,19,20,25]. This study primarily aimed to determine the clinical relevance of YAP1 by examining its association with key clinicopathologic parameters and pathological treatment response and to explore the impact of neoadjuvant chemotherapy on YAP1 expression by comparing its levels in pre- and post-treatment tumor tissues of patients with LABC.

Several previous studies have concluded with conflicting results regarding the prognostic and predictive role of YAP1 in breast cancer; Cha et al. demonstrated that high nuclear YAP1 expression was associated with HR negativity, increased Ki-67 index, lymph node metastasis, and inferior distant metastasis-free and disease-free survival (DFS), particularly in TNBC patients (HR for DFS: 3.208, *p* = 0.0105; HR for DMFS: 2.384, *p* = 0.0367) [21]. Similarly, Ding et al. reported that high nuclear and total YAP expression in primary breast tumors predicted worse DFS in TNBC and was identified as an independent prognostic factor in multivariate models [28]. In contrast, Cao et al. showed that YAP1 was mainly expressed in luminal A tumors and associated with favorable prognosis, including improved DFS in luminal subgroups [23]. These discrepancies may reflect subtype-specific roles of YAP1, as also highlighted in the meta-analysis by Li et al., which showed that YAP expression was significantly higher in TNBC compared to normal tissue (OR: 18.23, *p* < 0.001), while non-TNBC tumors exhibited lower YAP1 positivity (OR: 0.15, *p* < 0.001) [29]. In our study, we demonstrated that YAP1 positivity was significantly associated with axillary pathological complete response (*p* = 0.01), HER-2-enriched tumor subtype (*p* = 0.001), and high Ki-67 index (*p* = 0.028). YAP1 expression was most frequently detected in luminal/HER-2 and HER-2-only tumors and least in the luminal A subtype.

In terms of therapeutic response, the current evidence supports a potential role of YAP1 in chemoresistance across different tumor types. Yuan et al. demonstrated that YAP1 overexpression was associated with poor neoadjuvant chemotherapy response in TNBC, mediated via the MMP7/CXCL16 axis. In this model, YAP1 upregulation suppressed CXCL16-driven CD4+/CD8+ TIL recruitment and increased MMP7 expression, resulting in elevated RCB scores and reduced tumor remission [20]. Similarly, Vici et al. reported that while YAP or TAZ alone did not impact pathological response, the combined expression of YAP in both tumor and stromal compartments was independently associated with decreased likelihood of achieving pCR (OR: 7.13, *p* = 0.029) and shorter disease-free survival (HR: 3.07, *p* = 0.016) in TNBC patients [30]. Furthermore, in a chemohormonal setting, Matsuda et al. observed that elevated nuclear YAP1 in residual tumor tissue after neoadjuvant docetaxel-based therapy in prostate cancer was an independent predictor of biochemical recurrence [15]. On the other hand, a prior study by Isaogullari et al. failed to demonstrate a significant association between YAP and pCR [31]. In our study, while the association between YAP1 and breast pCR did not reach statistical significance, higher but not statistically significant pCR rates in YAP1-positive tumors were noted (44.4% vs. 17.1%, *p* = 0.07). The reason for not achieving pCR in breast tissue might be explained by the molecular heterogeneity and limited sample size. Another explanation might be the role of YAP1 in regulating cell motility, epithelial–mesenchymal transition (EMT), and cytoskeletal remodeling. YAP1-driven EMT programs and integrin signaling may enhance tumor cell plasticity and promote clearance of micrometastases, particularly from lymphatic compartments [32,33,34,35]. This mechanistic axis could partially explain the differential impact of YAP1 on axillary versus breast pCR observed in our cohort.

Breast pCR rates were highest in luminal/HER-2 (71.4%) and HER-2-positive (42.9%) subtypes, consistent with the distribution of YAP1 positivity and supporting its link with treatment response in these groups. These findings are in line with those reported by Goktas Aydin et al., who observed pCR rates of 3.7% in luminal A, 21.1% in luminal B/HER-2-positive, 24.7% in HER-2-positive/ER-negative, and 32.9% in TNBC subtypes (*p* < 0.001) [36]. Although not statistically significant (*p* = 0.06), breast pCR tended to be higher in premenopausal patients, suggesting a possible influence of hormonal status on treatment response.

Interestingly, in our study, YAP1 negativity was associated with more aggressive pathological features post NAC, including significantly higher rates of lymphatic and vascular invasion. This finding aligns with emerging evidence suggesting that nuclear YAP1 activity may exert a tumor-suppressive role by maintaining epithelial integrity. Cordenonsi et al. demonstrated that nuclear YAP1 preserves epithelial architecture, and its loss triggers epithelial-to-mesenchymal transition and enhances invasive behavior [16]. Similarly, studies by Barry et al. and Zhou et al. highlighted that cytoplasmic retention of YAP1 correlates with increased motility, invasion, and impaired cell–cell adhesion, promoting tumor progression [37,38].

Kim et al. showed that nuclear YAP expression was significantly correlated with high Ki-67 in colorectal adenocarcinoma (*p* = 0.002), while cytoplasmic YAP expression showed an inverse relationship [39]. In contrast, Cao et al. reported an inverse correlation between YAP and Ki-67 expression in breast cancer, where YAP positivity was more frequent in luminal A tumors with low proliferative index [23]. Our results also demonstrated a significant association between high Ki-67 index and YAP1 positivity (*p* = 0.028), suggesting a potential link between YAP1 activity and proliferative capacity in breast tumors. Due to the small sample size, this correlation was not observed in the post-treatment setting.

In our cohort, neoadjuvant chemotherapy led to a statistically significant decrease in nuclear YAP1 expression (*p* = 0.004) and a concomitant increase in cytoplasmic localization (*p* = 0.002), suggesting dynamic spatial modulation of YAP1 in response to treatment. This shift, however, is reversible: matrix stiffness, growth factor signaling, or taxane withdrawal can rapidly reactivate nuclear YAP1, which may reflect altered transcriptional activity and therapeutic vulnerability. Therefore, the post-NAC cytoplasmic pool likely represents an adaptive dormancy rather than permanent inactivation—a finding that supports therapeutic strategies aimed at blocking nuclear re-import or accelerating cytoplasmic degradation after chemotherapy.

Pharmacologic inhibitors such as verteporfin, which disrupt the YAP–TEAD interaction, have shown efficacy across multiple breast cancer subtypes [40], while upstream modulators like RNF187 have been proposed as potential targets in triple-negative disease [41].

The strong association between pre-treatment YAP1 expression and HER-2-positive subtype in our study further supports its relevance as both a biomarker of response and a candidate for molecular targeting, particularly in aggressive tumor types. When classified by molecular subtype, the nuclear-to-cytoplasmic shift in YAP1 expression was seen in most subgroups, but it was more noticeable in HER-2-positive tumors, which also had higher baseline YAP1 levels. Due to the small sample size, these subgroup trends were not statistically significant. However, the consistent directional change suggests a broader, treatment-driven modulation of YAP1 localization rather than an effect specific to a subtype.

YAP1 expression was associated with greater nodal clearance, particularly in HER-2-positive tumors. Although previous studies have linked YAP1 to chemoresistance and poor outcomes, especially in triple-negative or basal-like breast cancer, our findings suggest that YAP1 may exert context-dependent effects. In HER-2-positive tumors, YAP1 may enhance HER-2-driven signaling and increase chemosensitivity through PI3K/AKT/mTOR modulation [29,30]. Conversely, in TNBC, YAP1 overexpression has been associated with EMT and stem-like phenotypes that promote therapeutic resistance [31].

Despite the novel insights provided, our study has several limitations. First, the relatively small sample size, particularly for post-treatment surgical specimens (*n* = 30), may have limited the statistical power for some subgroup analyses and reduced the ability to draw definitive conclusions about post-treatment expression patterns. This study did not assess survival, which could represent another limitation. While post-treatment YAP1 expression could not be dichotomized due to the limited availability of residual tumor tissue, this did not undermine our primary aim, which was to evaluate the chemotherapy-induced change in YAP1 localization. The functional assays were not performed to confirm that cytoplasmic retention of YAP1 reflects transcriptional inactivation, nor were dynamic changes in YAP1 localization correlated with downstream target gene expression. However, if validated in larger cohorts, YAP1 immunohistochemistry could be integrated into routine diagnostic workflows utilizing pre-treatment core biopsy specimens. In HER-2-positive breast cancer, high YAP1 expression may serve as a surrogate marker of nodal chemosensitivity, potentially informing surgical decision-making or the intensity of axillary management.

## 5. Conclusions

Our findings suggest that YAP1 expression, particularly its subcellular localization, may serve as a promising predictive biomarker in patients with locally advanced breast cancer undergoing neoadjuvant chemotherapy. And the higher incidence of lymphovascular invasion in YAP1-negative tumors may indicate a distinct biological behavior of residual disease.

Furthermore, this study highlights YAP1 as a dynamic, context-dependent biomarker: high baseline nuclear expression predicts nodal chemosensitivity, while chemotherapy induces a nuclear-to-cytoplasmic shift that may be an adaptive, but reversible, transcriptional quiescence. Strategies that lock YAP1 in the cytoplasm or disrupt YAP1–TEAD could complement standard treatments, especially in tumors with high pre-treatment nuclear YAP1. Further functional studies are warranted to determine whether this shift correlates with reduced oncogenic transcriptional output and whether such changes can be therapeutically exploited in YAP1-expressing breast cancers.

## Figures and Tables

**Table 1:** Patient and tumor characteristics.

Characteristics (*N* = 50)	
Age, median (range)	49.5 (24–72)
Menopausal status, *n* (%)	
Premenopausal	24 (48)
Postmenopausal	26 (52)
Tumor location, *n* (%)	
Left	23 (46)
Right	27 (54)
Tumor size (mm)	28 (20–36)
Baseline T stage (clinical)	
T1	7 (14)
T2	20 (40)
T3	18 (36)
T4	5 (10)
Baseline N stage (clinical)	
N1	24 (48)
N2	15 (30)
N3	11 (22)
Molecular phenotype, *n* (%)	
Luminal type	27 (54)
HR-positive/HER-2-positive	7 (14)
HER-2-positive/HR-negative	7 (14)
Triple-negative	9 (18)
ER positivity, *n* (%)	34 (68)
PR positivity, *n* (%)	30 (60)
HER-2 positivity, *n* (%)	14 (28)
Baseline Ki-67, median (range)	35 (10–90)
Axillary pCR, *n* (%)	20 (40.0)
Breast pCR, *n* (%)	11 (22)
YAP1-positive expression (pre-NAC), *n* (%)	9 (18)
YAP1-negative expression (pre-NAC), *n* (%)	41 (82)

HR: hormone receptor; ER: estrogen receptor; PR: progesterone receptor; pCR: pathological complete response; NAC: neoadjuvant chemotherapy; YAP: Yes-associated protein.

**Table 2:** Summary of associations between pre-treatment YAP1 expression and clinicopathologic parameters.

Parameter	YAP1-Positive (*n =* 9)	YAP1-Negative (*n* = 41)	*p*
Axillary pCR, *n* (%)	7 (77.8)	13 (31.7)	0.021
Breast pCR, *n* (%)	4 (44.4)	7 (17.1)	0.093
Molecular Subtype, *n* (%)			0.001
Luminal (HR+/HER–2-)	1 (11.1)	26 (63.4)	
HR+/HER-2+	5 (55.6)	2 (4.9)	
HR−/HER-2+	1 (11.1)	6 (14.6)	
Triple-negative	2 (22.2)	7 (17.1)	
ER positivity, *n* (%)	7 (77.8)	33 (80.5)	NS
PR positivity, *n* (%)	6 (66.7)	30 (73.2)	NS
HER-2 positivity, *n* (%)	6 (66.7)	9 (22.0)	0.01
Ki-67 (Pre-NAC), median (%)	75	55	0.028

YAP: Yes-associated protein; pCR: pathological complete response; HR: hormone receptor; ER: estrogen receptor; PR: progesterone receptor; NAC: neoadjuvant chemotherapy; NS: not significant.

## Data Availability

The data that support the findings of this study are available from the corresponding author upon reasonable request.

## Abbreviations

LABC	locally advanced breast cancer
NAC	neoadjuvant chemotherapy
pCR	pathological complete response
YAP1	Yes-associated protein-1
TNBC	triple-negative breast cancer
IHC	immunohistochemistry
ER	estrogen receptor
HR	hormone receptor
PR	progesterone receptor
ROC	receiver operating characteristic
OR	odds ratio
DFS	disease-free survival
OS	overall survival
EMT	epithelial–mesenchymal transition
